# Imaging the snorkel effect during submerged germination in rice: Oxygen supply via the coleoptile triggers seminal root emergence underwater

**DOI:** 10.3389/fpls.2022.946776

**Published:** 2022-07-29

**Authors:** Katsuhiro Shiono, Akiko Koshide, Kazunari Iwasaki, Kazumasa Oguri, Takeshi Fukao, Morten Larsen, Ronnie N. Glud

**Affiliations:** ^1^Department of Bioscience and Biotechnology, Fukui Prefectural University, Fukui, Japan; ^2^HADAL and Nordcee, Department of Biology, University of Southern Denmark, Odense, Denmark; ^3^Research Institute of Global Change, Japan Agency for Marine-Earth Science and Technology, Yokosuka, Japan; ^4^Department of Ocean and Environmental Sciences, Tokyo University of Marine Science and Technology, Minato, Japan; ^5^Danish Institute of Advanced Studies, University of Southern Denmark, Odense, Denmark

**Keywords:** anoxia, flatbed scanning system, hypoxia, oxygen imaging, *Oryza sativa*, seminal root development, planar oxygen optode, spatiotemporal imaging

## Abstract

Submergence during germination impedes aerobic metabolisms and limits the growth of most higher plants. However, some wetland plants including rice can germinate under submerged conditions. It has long been hypothesized that the first elongating shoot tissue, the coleoptile, acts as a snorkel to acquire atmospheric oxygen (O_2_) to initiate the first leaf elongation and seminal root emergence. Here, we obtained direct evidence for this hypothesis by visualizing the spatiotemporal O_2_ dynamics during submerged germination in rice using a planar O_2_ optode system. In parallel with the O_2_ imaging, we tracked the anatomical development of shoot and root tissues in real-time using an automated flatbed scanner. Three hours after the coleoptile tip reached the water surface, O_2_ levels around the embryo transiently increased. At this time, the activity of alcohol dehydrogenase (ADH), an enzyme critical for anaerobic metabolism, was significantly reduced, and the coleorhiza covering the seminal roots in the embryo was broken. Approximately 10 h after the transient burst in O_2_, seminal roots emerged. A transient O_2_ burst around the embryo was shown to be essential for seminal root emergence during submerged rice germination. The parallel application of a planar O_2_ optode system and automated scanning system can be a powerful tool for examining how environmental conditions affect germination in rice and other plants.

## Introduction

Submergence during germination impedes aerobic metabolism and limits the growth of most higher plants (Pucciariello, 2020; Cho et al., 2021; Gómez-Álvarez and Pucciariello, 2022), many of which are commercially important crops (Biswas and Kalra, 2018; Zaman et al., 2018, 2019). Under normoxic conditions, gibberellic acid (GA) promotes germination by activating the starch-degrading enzyme α-amylase (Bethke et al., 1997). However, low O_2_ conditions inactivate GA signaling (Benech-Arnold et al., 2006; Hoang et al., 2013; Gómez-Álvarez and Pucciariello, 2022) and suppress the expression of GA-dependent α-amylase (Perata et al., 1992; Loreti et al., 2003). Thus, for most plants, anaerobic conditions prevent starch degradation in the endosperm, which, in turn, prevents germination. However, rice, a wetland plant, can germinate during submergence because it activates a GA-independent α-amylase (i.e., subfamily 3 of α-amylase) under low O_2_ conditions for starch-degradation (Perata et al., 1992). The expression of GA-independent α-amylase is promoted by hypoxia-dependent Ca^2+^ signals that are regulated by calcineurin B-like protein (CBL)-interacting protein kinase15 (CIPK15) (Lee et al., 2009; Ho et al., 2017). Germination under submergence relies on maintaining high α-amylase activity under apparent anaerobic conditions (Miro and Ismail, 2013; Cho et al., 2021; Gómez-Álvarez and Pucciariello, 2022).

The seeds of wetland plants such as some *Echinochloa* species (rice-like weeds) and rice can rapidly elongate a hollow coleoptile under submerged conditions (Atwell et al., 1982; Alpi and Beevers, 1983; Kawai and Uchimiya, 2000; Yamasue, 2001). The rapid growth is due to both cell division (at least initially) and cell elongation (Takahashi et al., 2011). Coleoptile length under submergence is positively regulated by auxin and by translocation of auxin via the influx carrier AUXIN TRANSPORT1 (AUX1) (Nghi et al., 2021). Once the coleoptile reaches the water surface, it is thought to generate a passage by which O_2_ can reach the underwater organs and enable respiration (Pucciariello, 2020). Rapid coleoptile elongation could thus represent a strategy to circumvent ambient anoxia (Narsai et al., 2015) and has been referred to as the “snorkel effect” (Kordan, 1974). Soon after the coleoptile reaches the water surface, a seminal root forms and gradually elongates (Kordan, 1974; Hoshikawa, 1989). It has thus been speculated that O_2_ acquisition via the coleoptile is essential for seminal root emergence in rice. However, this hypothesis has never been confirmed or resolved by direct measurements, and the spatiotemporal O_2_ dynamics around germinating rice remain unclear.

The planar O_2_ optode is a semitransparent optical sensor that allows real-time and two-dimensional imaging of O_2_ using luminescent or fluorescent indicators of molecular oxygen (Santner et al., 2015; Li et al., 2019). Planar O_2_ optode systems have enabled imaging of the spatiotemporal O_2_ dynamics within benthic communities (Glud et al., 1996; Wenzhöfer and Glud, 2004) and have later been used to observe the oxic halo around individual plant roots in the sediment and soil (Frederiksen and Glud, 2006; Blossfeld and Gansert, 2012; Larsen et al., 2015; Li et al., 2019; Maisch et al., 2019; Martin et al., 2019). While a planar optode reveals the O_2_ distribution within the sediment, a vertically positioned flatbed scanner can concurrently monitor spatial and temporal root development (Dannoura et al., 2008, 2012).

The aim of this study was to test the snorkel hypothesis, i.e., that O_2_ acquisition via the coleoptile initiates seminal root emergence, by obtaining images of a germinating submerged rice seed with high spatiotemporal resolution. Specifically, we monitored (i) seed germination dynamics with an automated scanning system, (ii) O_2_ dynamics with the planar O_2_ optode system and (iii) the activity of an anaerobic marker enzyme, ADH, in the embryo. Our measurements confirm previous speculation that O_2_ transported by the coleoptile initiates seminal root emergence.

## Materials and methods

### Plant material and culture

Rice (*Oryza sativa* L. cv. Nipponbare) was used in this study. Seeds were sterilized for 30 min in 0.6% (w/v) sodium hypochlorite and washed thoroughly with deionized water. In all experiments, rice plants were grown in a controlled-environment room in the dark (26°C, relative humidity over 50%).

**FIGURE 1:**
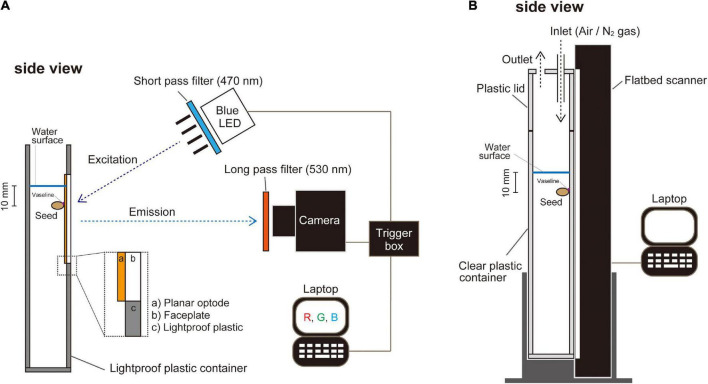
A schematic representation of the plant growth container in front of a color-ratiometric planar O_2_ optode system and the growth monitoring system. **(A)** The planar O_2_ optode, which is cast on an optical and the O_2_ sensitive chemistry, is excited by a blue LED (λ-peak = 457 nm) with a 470 nm short-pass filter. Emission images are captured by a digital camera with a 530 nm long-pass filter. **(B)** Setup of monitoring the growth development by an automated scanning system. Images of A4 size (210 mm × 297 mm) were recorded every 20 min. Oxygen availability in the experimental setup was controlled by an influx of air (normoxic atmosphere) or N_2_ gas (anoxic atmosphere).

For planar O_2_ optode experiments, we used a growth container (44 mm × 80 mm × 200 mm) made of opaque polyvinyl chloride with an inlaid of a faceplate courted with an O_2_-sensitive optode cocktail (see below *Oxygen imaging by planar optode system*) (Figure 1A). To monitor germination in normoxic or anoxic atmospheres, we used a clear plastic container (25 mm × 220 mm × 300 mm high) that was placed in contact with a vertically positioned flatbed scanner (GT-S620, Seiko Epson, Suwa, Japan) (see below *Monitoring plant growth*) (Figure 1B). Seeds were attached to the optode faceplate or wall of the clear plastic container with Vaseline (Nacalai Tesque, Kyoto, Japan) (Figures 1A,B). Subsequently, a stagnant deoxygenated nutrient solution was added to the containers to a level 10 mm above the seeds (Figures 1A,B).

The nutrient solution contained 0.47 mM K_2_SO_4_, 0.38 mM CaSO_4_, 0.55 mM Ca(NO_3_)_2_, 0.1 mM MgSO_4_, 78 μM (NH_4_)_2_SO_4_, 50 μM KH_2_PO_4_, 25 μM Na_2_SiO_3_, 12.5 μM Fe(III)-EDTA, 12.5 μM KCl, 6.25 μM H_3_BO_3_, 0.5 μM MnSO_4_, 0.5 μM ZnSO_4_, 250 nM NiSO_4_, 125 nM CuSO_4_, and 125 nM Na_2_MoO_4_. In addition, the solution contained 625 μM MES, and pH was adjusted to 6.5 using KOH. The solution was made stagnant by adding 0.1% (w/v) dissolved agar (Wiengweera et al., 1997) and deoxygenated by flushing it with N_2_ gas flushing for 15 min. The dilute agar prevents convective movements and mimics the changes in gas composition in waterlogged soils (e.g., decreased O_2_, increased ethylene) (Wiengweera et al., 1997). The solution remained hypoxic throughout the experiment (O_2_ concentration < ca. 30 μM).

### Oxygen imaging by planar optode system

In the present study, we used a color ratiometric planar O_2_ optode system described by Larsen et al. (2011), in which O_2_ concentration is calculated from the ratios of the intensities of red and green in a single digital image of RGB color. PtTFPP [Platinum (II)-5,10,15,20-tetrakis-(2,3,4,5,6-pentafluorophenyl)-porphyrin] is commonly used for planar O_2_ optodes as an O_2_-sensitive indicator (Lee and Okura, 1997; Oguri et al., 2006). PtTFPP has a peak emission at 650 nm (Larsen et al., 2011). Therefore, the red signal in the RGB color image is derived from PtTFPP. A coumarin dye, Macrolex^®^ fluorescence yellow 10GN (MY), is not quenched by O_2_ and its luminescence intensity is unaffected by the O_2_ concentration. MY has the peak emission wavelength at 480 nm in the polystyrene matrix (Larsen et al., 2011). Therefore, the green signal in the RGB color image derived from MY emission serves as an internal reference. To obtain the O_2_ concentration image, the ratiometric calculation is conducted on each pixel of the optode faceplate (Larsen et al., 2011).

A faceplate (75 mm × 50 mm × 3 mm thickness, fiber optic faceplates #55-142, Edmund Optics, Barrington, NJ, United States) was immersed in 10% (v/v) dichloro-dimethyl-silane (Sigma-Aldrich, St. Louis, MO, United States) in 99.8% toluene (Sigma-Aldrich) for 2 h, in order to enhance adhesion of sensor cocktail to the faceplate. The faceplate was washed with 99.5% ethanol (FUJIFILM Wako Pure Chemical, Osaka, Japan) and dried at 110°C overnight. We prepared a sensor cocktail which contained 10% (w/v) polystyrene granules (Goodfellow, Huntingdon, United Kingdom), 0.2% (w/v) PtTFPP (Frontier Scientific Inc., Logan, UT, United States), 0.4% (w/v) MY and 1.5% (w/v) titanium dioxide (DuPont, Wilmington, DE, United States) in 99.8% toluene and mixed it with heating at 90°C. The faceplate was thinly coated with the sensor cocktail and dried at room temperature overnight. The edge of the optode faceplate (i.e., planar O_2_ optode) was attached to the plastic container wall with silicon glue (Figure 1A). Here we note that no toxic effects of planar optode have been reported.

To excite the O_2_-sensitive luminophores, we applied a high luminous efficacy blue LED (λ-peak = 457 nm, LZ1-00B202, Osram Sylvania Inc., Wilmington, NC, United States) in combination with a 470 nm short-pass filter (Blue dichroic color filter, CDB-5051, UQG Optics Ltd., Cambridge, United Kingdom) (Figure 1A). Emission was captured with a digital single-lens reflex camera (Canon EOS Kiss F, Canon, Tokyo, Japan) equipped with a macro lens (Sigma 50 mm F2.8 EX DG Macro, Sigma, Kawasaki, Japan) and a 530 nm long-pass filter (Schott OG-530, Schott AG, Mainz, Germany) (Figure 1A). The camera was modified by removing its near-infrared (NIR) filter to increase the camera’s sensitivity to red light. The camera was connected to a computer and controlled with the software Look@RGB,^1^ which enables the splitting of images into red, green, and blue channels (Figure 1A). The intensity ratio, defined as (*red channel* - *green channel*)/*green channel* (Larsen et al., 2011), was used to derive the O_2_-dependent signal at the surface of the optode. To obtain calibration curves, water was deoxygenated by flushing it with N_2_ gas for 30 min. The dissolved oxygen (DO) level was about 0.7% of air saturation at 26°C. The water was then gradually oxygenated by air flushing while concurrently acquiring O_2_-sensitive images and recording the ambient O_2_ concentration with a DO meter (Seven2Go™ pro DO meter S9, Mettler Toledo, Greifensee, Switzerland). In total, we collected about 10 calibration-level points up to 100% air saturation in the water for calibration. Finally, an anoxic (0% DO) image was captured by adding about 0.4 g of sodium dithionate (Na_2_S_2_O_4_, FUJIFILM Wako Pure Chemical) to the water (250 ml). Calibration calculations and image processing were done as described by Larsen et al. (2011). All images were processed using ImageJ (version 1.52a).^2^ O_2_ concentration was expressed as % air saturation. At 26°C, 100% air-saturation in freshwater is equivalent to 254 μM of O_2_.

### Monitoring plant growth

The growth container was placed in contact with a vertically positioned flatbed scanner. The container was closed with a plastic lid (Figure 1B). O_2_ levels within the enclosed atmosphere were controlled by continuously flushing with air (normoxic atmosphere) or N_2_ gas (anoxic atmosphere) (Figure 1B). Growth images (600 dpi, 24-bit color) were captured by the flatbed scanner every 20 min using application software to schedule mouse operations on a computer (UWSC 5.3.0.2).^3^ The automated scanning system has a broader capture view (297 mm × 210 mm) that is broader than the capture views of our O_2_ planar optode systems (i.e., 75 mm × 50 mm) (Figure 1A). This makes it possible to monitor more replications under various conditions and collect samples at the proper timing.

### Anatomical observations

For embryo observations, seeds were embedded in a cryo-embedding medium (SCEM, Leica Biosystems, Wetzlar, Germany) and frozen in liquid nitrogen. The specimen block was cut into 10-μm sections with a CM-3050S cryostat (Leica Biosystems). We used the Kawamoto cryosectioning method (Chiou et al., 2018) in which the chamber was set at –15°C and the object holder was set at –25°C. The sections were gently mounted on slides. Cross-sections were photographed with a light microscope (Axio Imager.A2, Carl Zeiss, Oberkochen, Germany) equipped with a CCD camera (AxioCam MRc CCD, Carl Zeiss). For coleoptile observations, coleoptiles at 3–7 mm behind the embryo were embedded in 5% agar, and 100 μm sections were made using a vibrating microtome (Leica VT1200S; Leica Biosystems). Cross-sections were photographed with the above microscope. Coleoptile and seed semblances were photographed with a stereomicroscope (Leica M205FA, Leica Microsystems) with a CCD camera (Leica DFC7000T, Leica Microsystems).

### Alcohol dehydrogenase activity

Alcohol dehydrogenase (ADH) activity in embryos was assayed with the spectrophotometric method of Rumpho and Kennedy (1981). Embryos (40–84 mg fresh weight per sample) were separated from the endosperm and coleoptile with a razor, frozen in liquid nitrogen, ground in liquid nitrogen in a mortar and pestle, suspended in 0.5 ml fresh extraction buffer [100 mM Tris-HCl (pH 9.0), 20 mM MgCl (Nacalai Tesque), 0.1% (v/v) 2-mercaptoethanol (FUJIFILM Wako Pure Chemical)], and centrifuged at 13,000 × g for 15 min at 4°C. Ten microliter of the supernatant was mixed with 255 μl assay buffer [58 mM Tris-HCl (pH 9.0), 1.12 mM β-NAD^+^ (Oriental yeast, Tokyo, Japan)] and 15 μl ethanol. For the control, 15 μl of water was used instead of ethanol. We then quickly measured the change in A_340_, which is related to NADH concentration, for 1 min at 26°C, by a microplate reader (SpectraMax iD3, Molecular Devices, San Jose, United States). The enzyme activity was calculated by subtracting the reference activity from the sample activity and expressing ADH activity as the amount of NADH produced in 1 min (i.e., μmol g FW^–1^ min^–1^).

### Statistical analysis

The lengths of roots or shoots grown in normoxic and anoxic atmospheres were compared with two-sample *t*-tests at the 5% probability level. ADH activities were compared with one-way analysis of variance (ANOVA) and Tukey’s honest significant difference (HSD) for multiple comparisons at the 5% probability level. All statistical analyses were conducted using IBM SPSS Statistics version 25 (IBM, Armonk, NY, United States).

## Results

### Seminal root emergence requires a normoxic atmosphere

**FIGURE 2:**
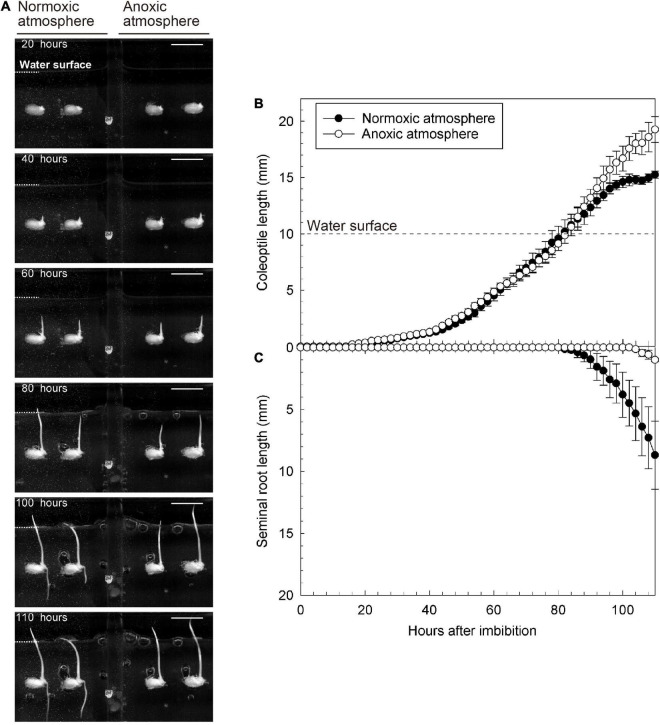
Growth of coleoptile and seminal root during submerged germination with normoxic or anoxic atmosphere. **(A)** A sequence of the submerged germination processes. Bars = 10 mm. **(B)** Coleoptile length, and **(C)** seminal root length under germination. Means ± SE, *n* = 4–5. The time lap images of submerged germination are shown in Supplementary Video 1.

Submerged seeds were placed in anoxic and normoxic conditions, respectively, and in both treatments, a coleoptile emerged and elongated 25 h after imbibition, without any seminal root emergence (left panel of Figure 2A and Supplementary Video 1). However, as the coleoptile reached the water surface, 80–85 h after imbibition, seminal roots began to emerge in the normoxic treatment (left panel of Figures 2A–C and Supplementary Video 1) and the elongation rate of coleoptiles drastically slowed down from 0.25 to 0.18 mm/h (Figure 2B). In contrast, a seminal root did not emerge in the anoxic treatment, although the coleoptile tip was above the water surface (right panel of Figure 2A and Supplementary Video 1). Additionally, the elongation rates of the coleoptile both underwater and above water remained constant (0.21 and 0.34 mm/h, respectively) (Figure 2B).

**FIGURE 3:**
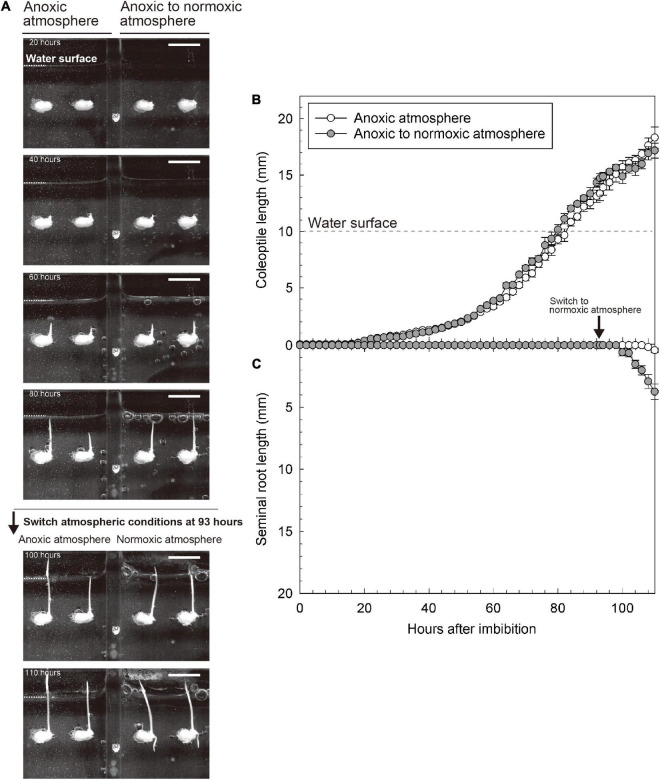
Reaction of coleoptile and seminal root growth following the switching from anoxic to normoxic atmospheres. **(A)** An image sequence of the submerged germination processes. Bars = 10 mm. **(B)** Coleoptile length and **(C)** seminal root length during germination. Means ± SE, *n* = 3–5. At 93 h after imbibition, the atmospheric conditions were switched from anoxic to normoxic. The time lap images of growth responses following the switching from an anoxic to a normoxic atmosphere are shown in Supplementary Video 2.

To further explore the importance of O_2_ in the overlying atmosphere for root emergence, we switched the atmospheric conditions from anoxic to normoxic at 93 h after imbibition. At that time, the coleoptile tip had emerged from the water surface, but seminal roots were not observed (Figure 3). Eight hours after the switch, seminal roots appeared (right panel of Supplementary Video 2). Seminal roots were well developed at 110 h after imbibition (i.e., 17 h after the switch) (at 110 h in Figures 3A,C). However, no roots were observed in a parallel setup where we maintained an anoxic atmosphere (at 110 h in Figures 3A–C and Supplementary Video 2) demonstrating that atmospheric O_2_ was essential for seminal root emergence.

### Spatiotemporal oxygen dynamics during coleoptile development

**FIGURE 4:**
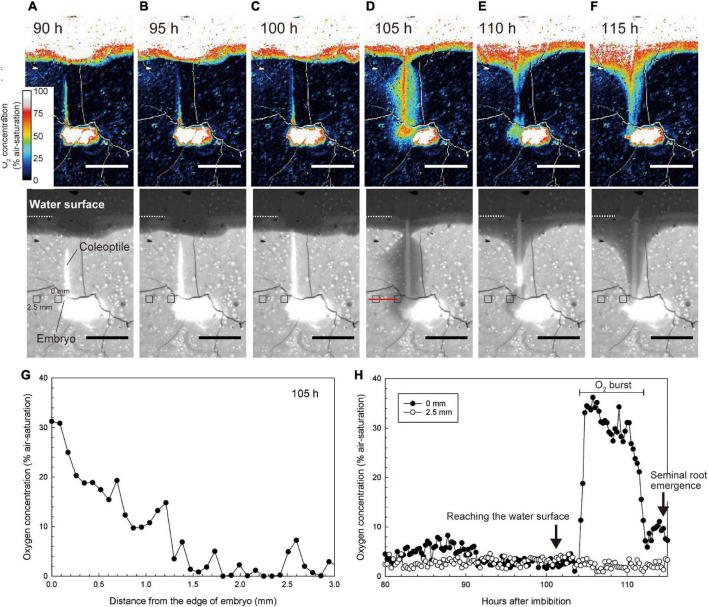
Spatiotemporal O_2_ dynamics under normoxic atmosphere. **(A–F)** An image sequence of the submerged germination process as recorded by the planar O_2_ optode system. Bars = 10 mm. Upper color images are O_2_ concentration images. Lower monochrome images are red images to visualize the growth of coleoptile. At the 101 h after imbibition, the coleoptile tip reached the water surface. The coleoptile tip was submerged in **(A–C)** or above water in **(D–F)**. Note: the O_2_ value on the seeds and the coleoptile erroneously appear constantly high (i.e., over 50–100% air-saturation) due to a scattering artifact reflecting the white colors of seeds and coleoptiles. **(G)** O_2_ gradient toward the embryo at 105 h after imbibition (i.e., along the red line in the lower panel of **D**) when a transient O_2_ burst was observed at 105 h after imbibition. **(H)** Dynamics of O_2_ concentration at 0 mm and 2.5 mm from the embryo (e.g., small squares in the lower panels of **A–F**). The sequence of O_2_ images during submerged germination is shown in Supplementary Video 3.

Before the coleoptile tip reached the water surface, O_2_ was deficient around the embryo and coleoptile [below 8% (air-saturation), Figures 4A–C and Supplementary Video 3]. Interestingly, when the coleoptile tip reached the water surface, the O_2_ level around the embryo almost instantaneously increased to 30–40% air saturation (Figure 4D and Supplementary Video 3). The oxygen-rich halo extended 1.5 mm away from the embryo (Figure 4G). O_2_ levels gradually fell to background levels in about 8 h (Figure 4H). Our observations of seven seeds showed that the transient O_2_ burst occurred 3.2 ± 2.0 h after the coleoptile tip reached the water surface (mean ± standard deviation). Then, seminal root emergence was initiated about 9.6 ± 1.5 h after the transient O_2_ burst.

In Figures 4A–F, the O_2_ value on the seeds and the coleoptile appeared to be over 50–100% air-saturation, but this was an artifact due to the scattering of light off the seeds and coleoptiles.

**FIGURE 5:**
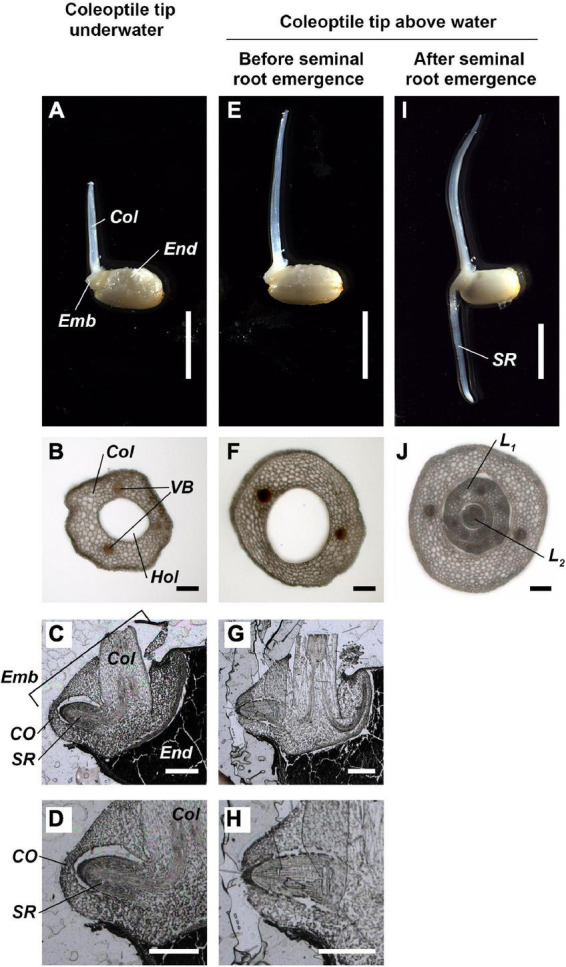
Anatomy of coleoptile and embryo during submerged germination below a normoxic atmosphere. **(A–D)** The coleoptile tip was underwater. **(E–H)** The coleoptile tip was above water, but seminal roots have not yet emerged. **(I,J)** The coleoptile tip was above water and after seminal root emergence. Cross-sections of the coleoptile at the 5 mm from the embryo **(B,F,J)**. Cross-sections of the embryo **(C,D,G,H)**. *Col*, coleoptile; *CO*, coleorhiza; *Emb*, embryo; *End*, endosperm; *Hol*, hollow; *L*_1_, 1st leaf; *L*_2_, 2nd leaf; *SR*, seminal root; *VB*, vascular bundles. Scale bars: 5 mm in **(A,E,I)**; 100 μm in **(B,F,J)**; 500 μm in **(C,D,G,H)**.

The seminal roots did not emerge while the tip of hollow coleoptile was submerged (Figures 5A,B) and remained enveloped by coleorhiza inside the embryo (Figures 5C,D). However, as the hollow coleoptile (Figure 5F) reached the water surface (Figure 5E), the seminal root broke the coleorhiza and emerged from the embryo (Figures 5G,H). Between 24 and 33 h after the coleoptile tip reached the water surface, the seminal root elongate d about 10-mm-length (Figure 5I). At this time, the hollow space of the coleoptile was filled by the first and second leaves (Figure 5J). Aerenchyma had still not formed in the coleoptile or in the first or second leaves (Figure 5J).

### Alcohol dehydrogenase enzyme activity in the embryo

**FIGURE 6:**
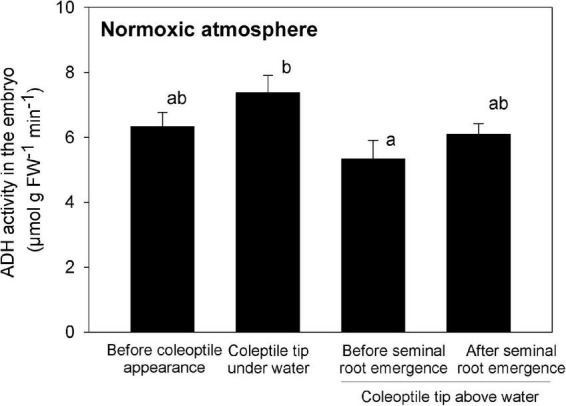
ADH activity in the embryo during submerged germination below a normoxic atmosphere. Mean ± SE. *n* = 3 or 4. Different lowercase letters denote significant differences among four different growth phases (*P* < 0.05; one-way ANOVA, and then Tukey HSD test for multiple comparisons).

Before the formation of the coleoptile and while the tip of the evolving coleoptile remained submerged, the ADH activity in the embryo was high (Figure 6), consistent with an anaerobic environment. However, as the coleoptile emerged from the water surface, the ADH activity was significantly reduced, reaching a minimum of 5.3 μmol g FW^–1^ min^–1^ (Figure 6), implying O_2_ levels were improved in the embryo. However, the ADH activity increased slightly (but not significantly) after the seminal root had emerged from the embryo (right bar in Figure 6), possibly as a result of increased O_2_ consumption.

## Discussion

**FIGURE 7:**
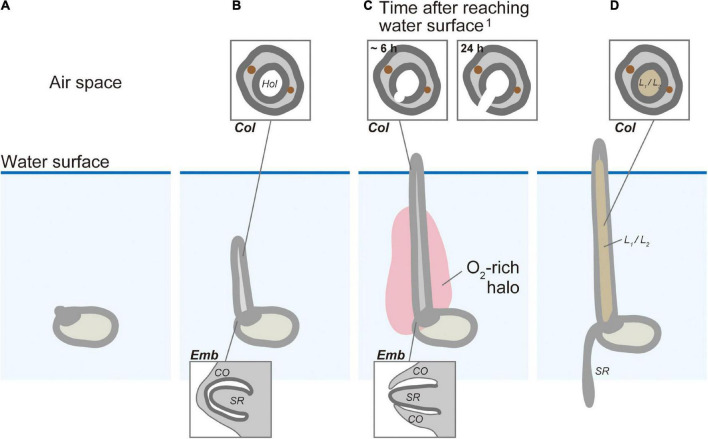
The conceptual model of the snorkel effect during submerged rice germination. **(A)** Before coleoptile appearance. **(B)** Coleoptile tip underwater. **(C)** Coleoptile tip above water and before seminal root emergence. **(D)** Coleoptile tip above water and after seminal root emergence. *Col*, coleoptile; *CO*, coleorhiza; *Emb*, embryo; *Hol*, hollow; *L*_1_, 1st leaf; *L*_2_, 2nd leaf; *SR*, seminal root.^1^ The timing of cell death after aeration was predicted based on previous research by Kawai and Uchimiya (2000).

The real-time monitoring of the submerged germination and the associated O_2_ distribution that we observed in this study provides a detailed look at the spatiotemporal dynamics and coupling of crucial processes in the germination process and its overall linkage to O_2_ availability in rice. Submerged rice germination occurred in an orderly progression of rooting after the coleoptile reached the water surface (Figure 7). Almost 50 years ago, Kordan (1974) hypothesized that the coleoptile acts as a snorkel to acquire atmospheric oxygen to initiate elongation of the first leaf and seminal root emergence. However, this hypothesis has never been confirmed by direct measurements of O_2_ transport. We visualized the snorkel-like behavior of the coleoptile, resulting in the transient O_2_ burst around the embryo. This O_2_ burst is required for seminal root emergence underwater.

### A transient oxygen burst around the embryo triggers seminal root emergence

Seminal root primordia were unable to break through the coleorhiza without transient oxygenation at the embryo (Figures 5C,D). When a fully developed coleoptile reached the water surface, a burst in O_2_ availability at the embryo was observed and seminal root induction occurred within 10 h (Figures 4H, 5G,H). Seminal root emergence was previously reported to occur within 1 day after aeration of submerged rice seeds at 30°C (Narsai et al., 2017), but the present study shows that this can happen much faster, even at lower temperatures (26°C).

Unlike the development of seminal roots, the development of adventitious roots of rice emerging at the stem nodes under submerged conditions appears to be promoted by hypoxia (Lorbiecke and Sauter, 1999; Steffens and Rasmussen, 2016). The ability of nodal adventitious root primordia to break through the overlying epidermal cells has been linked to elevated concentrations of ethylene (Lorbiecke and Sauter, 1999; Steffens et al., 2006). Ethylene signaling mediated by reactive oxygen species induces programmed cell death (PCD) in the nodal epidermis above adventitious root primordia (Mergemann and Sauter, 2000; Steffens et al., 2012). This assures that the tip of the growing root is not damaged during emergence (Mergemann and Sauter, 2000; Steffens and Rasmussen, 2016). Both epidermal PCD and adventitious root growth are promoted by ethylene and gibberellin but are inhibited by abscisic acid (Steffens and Sauter, 2005; Steffens et al., 2006). The hormonal network and molecular mechanisms that initiate seminal root emergence under aerobic conditions are unclear. Further research on how hormones regulate seminal root development are needed to more fully understand how seedling adapt to submergence.

### A conceptual model for the snorkel effect under submerged germination in rice

Based on previous investigations and the present results, we propose a germination model in which transient oxidation triggers root emergence (Figure 7). Germination of submerged rice (Figure 7A) induces a gradual elongation of a hollow coleoptile (Figures 5B, 7B). Seminal root primordia are trapped inside the coleorhiza in the embryo (Figures 5C,D, 7B). About 3 h after the coleoptile tip reaches the atmosphere, a transient O_2_-rich halo along the coleoptile and close to the embryo can be observed (Figures 4D,H, 7C). Kawai and Uchimiya (2000) observed that 6 h after the initial O_2_ exposure, cell death becomes visible on the adaxial surface in the coleoptile and a full opening is completed within 24 h (Kawai and Uchimiya, 2000). At this stage, the first leaf has still not elongated inside the coleoptile, and there is no aerenchyma in the coleoptile (Figures 5F, 7C), but the hollow coleoptile facilitates a continued O_2_ supply to the embryo. The reduction in ADH activity in the embryo after the coleoptiles emerge from the water surface (Figure 6) reflects an increase in the internal oxygen level within rice seedlings. However, the reduction in ADH activity was limited, probably because the internal oxygen concentration was still lower than that in air, keeping most parts of seedlings remained under moderate hypoxia. Subsequently, the leaves elongate inside the coleoptile (Figures 5J, 7D). The first leaf emerges at the site of a split in the coleoptile, resulting from PCD (Kawai and Uchimiya, 2000). The first leaf develops aerenchyma, which supports root aeration (Hoshikawa, 1989; Kawai and Uchimiya, 2000).

### Potential applications of automated scanning systems and planar oxygen optode

Many attempts have been made to directly sow rice seeds in the field to reduce the labor of transplanting seedlings from a nursery (Farooq et al., 2011). A better understanding of O_2_ dynamics around the seeds might help to explain why most of these attempts have failed. The current study demonstrates that an automated scanning system with a planar O_2_ optode could visualize the dynamics of O_2_ and concurrent seedling growth at very high spatiotemporal resolution. Our approach might facilitate and improve further studies of direct seeding methods (e.g., wet seeding, water seeding, dry seeding with/without chemical coating) for rice as well as other crops and plant species.

## Data availability statement

The original contributions presented in this study are included in the article/Supplementary material, further inquiries can be directed to the corresponding author.

## Author contributions

KS conceived the study. KS, ML, RG, and TF designed the experiments. KS, AK, and KI performed most of the experiments and analyzed the data. KS established the optode system supported with KO, ML, and RG. KS drafted the manuscript with ML, KO, RG, and TF contributions. All authors interpreted data, edited the manuscript, and approved the final manuscript.

## Footnotes

1
imaging.fish-n-chips.de

2
https://rsbweb.nih.gov/ij/

3
https://www.vector.co.jp/soft/winnt/util/se115105.html

## Supplementary material

Supplementary Video 1 Time-lapse images showing the germination process of submerged rice with normoxic or anoxic atmospheres.

Supplementary Video 2 Time-lapse images of the growth process following a shift from an anoxic to a normoxic atmosphere. At 93 h after imbibition, the atmospheric conditions of the right compartment were switched from anoxic to normoxic. The left compartment was kept anoxic throughout the experiment.

Supplementary Video 3 Time-lapse images of O_2_ distribution during germination of submerged rice. The rice plants in the middle image are the same target in Figure 4. The O_2_ value on the seeds and the coleoptile appeared to be over 50–100% air saturation, but this was an artifact due to the scattering of light off the seeds and coleoptiles.
